# Minimal and maximal lengths of quantum gravity from non-hermitian position-dependent noncommutativity

**DOI:** 10.1038/s41598-022-21098-3

**Published:** 2022-11-30

**Authors:** Latévi M. Lawson

**Affiliations:** 1 African Institute for Mathematical Sciences (AIMS) Ghana, Summerhill Estates, East Legon Hills, Santoe, P.O. Box LG DTD 20046, Legon, Accra, Ghana; 2 Laboratoire de Physique des Matériaux et des Composants à Semi-Conducteurs, Departement de Physique, Faculté des Sciences, Université de Lomé, 01 BP 1515, Lomé, Togo

**Keywords:** Physics, Quantum physics, Quantum mechanics

## Abstract

A minimum length scale of the order of Planck length is a feature of many models of quantum gravity that seek to unify quantum mechanics and gravitation. Recently, Perivolaropoulos in his seminal work (Perivolaropoulos in Phys. Rev. D 95:103523, 2017) predicted the simultaneous existence of minimal and maximal length measurements of quantum gravity. More recently, we have shown that both measurable lengths can be obtained from position-dependent noncommutativity (Lawson in J. Phys. A Math.Theor. 53:115303, 2020). In this paper, we present an alternative derivation of these lengths from non-Hermitian position-dependent noncommutativity. We show that a simultaneous measurement of both lengths form a family of discrete spaces. In one hand, we show the similarities between the maximal uncertainty measurement and the classical properties of gravity. On the other hand, the connection between the minimal uncertainties and the non-Hermicity quantum mechanic scenarios. The existence of minimal uncertainties are the consequences of non-Hermicities of some operators that are generators of this noncommutativity. With an appropriate Dyson map, we demonstrate by a similarity transformation that the physically meaningfulness of dynamical quantum systems is generated by a hidden Hermitian position-dependent noncommutativity. This transformation preserves the properties of quantum gravity but removes the fuzziness induced by minimal uncertainty measurements at this scale. Finally, we study the eigenvalue problem of a free particle in a square-well potential in these new Hermitian variables.

## Introduction

The idea of noncommutativity of space-time might provide deep indications about the quantum nature of space-time at a very small distance, where a full theory of quantum gravity must be invoked, has its root in string theory^1^. In fact, the noncommutativity of space-time is one of the promising candidate theories to the unification of quantum theory and General Relativity (GR). All the other candidate theories of unification such as string theory^2^, black hole theory^3^, loop quantum gravity^4^ predicted the existence of minimal measurement of quantum gravity at the Planck scale. To theoretically realize this minimal length scale in quantum mechanics, one has introduced a simple model, the so-called Generalized Uncertainty Principle (GUP)^5–7^ which is a gravitational correction to quantum mechanics. Mostly, these theories of quantum gravity are restricted to the case where there is a nonzero minimal uncertainty in the position. Only Doubly Special Relativity (DSR) theories^8–10^ suggest an addition to the minimal length, the existence of a maximal momentum. Recently, Perivolaropoulos proposed a consistent algebra that induces for a simultaneous measurement, a maximal length and a minimal momentum^11^. In this approach, the maximal length of quantum gravity is naturally arisen in cosmology due to the presence of particle horizons. Perivolaropoulos also predicted the simultaneous existence of maximal and minimal position uncertainties. More recently, we have shown that both position uncertainties can simultaneously be obtained from position-dependent noncommutativity and the minimal momentum is provided by the position-dependent deformed Heisenberg algebra^12^. In continuation of this work, we show that both lengths can also be derived from non-Hermitian position-dependent noncommutativity. The simultaneous presence of both lengths at this scale form a lattice system in which each site represented by the minimal length is separated by the maximal length. At each minimal length point results of the unification of magnetic and gravitational fields. As has been recently shown^13^, the maximal length allows probing quantum gravitational effects with low energies and manifests properties close to the classical ones of General Relativity (GR).

It is well known that the existence of minimal uncertainties in quantum mechanics induces among other consequences^14–18^ a non Hermicity of some operators that generate the corresponding Hilbert space^5,19–21^. In the present case, the minimal length in the X-direction and the minimal momentum in the $P_y$-direction lead to the non-Hermiticity of operators ${\hat{X}}$ and ${\hat{P}}_y$ that generate the noncommutative space^12^. Consequently, Hamiltonians ${\hat{H}}$ of systems involving these operators will in general also not be Hermitian. The corresponding eigenstates no longer form an orthogonal basis and the Hilbert space structure will be modified. In order to map these operators into their Hermitian counterparts. We introduce a positive-definite Dyson map $\eta$^22^ and its associated metric operator $\rho$ which generate a hidden Hermitian position-dependent noncommutativity by means of similarity transformation of the non-Hermitian one i.e $\eta \left( {\hat{X}},{\hat{Y}},{\hat{P}}_x,{\hat{P}}_y,{\hat{H}} \right) \eta = \left( {\hat{x}},{\hat{y}},{\hat{p}}_x,{\hat{p}}_y,{\hat{h}} \right) =\left( {\hat{x}}^\dag ,{\hat{y}}^\dag ,{\hat{p}}_x^\dag ,{\hat{p}}_y^\dag ,{\hat{h}}^\dag \right)$. Doing so, we tie a connection between the quantum mechanic noncommutativity with GUP^23–32^ and the non-Hermiticity quantum mechanic scenarios^33–47^. Furthermore, this transformation preserves the uncertainty measurements at this scale but removes the fuzziness induced by the minimal uncertainty measurements. Finally, within this hidden Hermitian space, we present the eigensystems of a free particle in a box. We show that the existence of maximal length induces strong quantum gravitational effects in this box. These effects are manifested by the deformations of quantum energy and these deformations are more pronounced as one increases the quantum levels, allowing the particle to jump from one state to another with low energies and with high probability densities^13^. These properties are similar to the classical gravity of General relativity where the gravitational field becomes stronger for heavy systems that curve the space, enabling the surrounding light systems to fall down with low energies. The resulting time inside of this space runs out more slowly as the gravitational effects increase.

In what follows, we explore in section 2, the similarities between our recent deformed noncommutativity with GUP and the pseudo-Hermiticity quantum mechanic scenarios. We show that these deformations lead to a non Hermiticity of the position operator ${\hat{X}}$ and the momentum operator ${\hat{P}}_y$. By constructing a Dyson map^22^ we provide their corresponding set of Hermitian counterparts. As a consequence of these deformations, we derive in section 3, the uncertainty measurements resulting from these deformations. In section 4, we study in terms of our new set of variables, the model of particles in a 2D box. We present our conclusion in section 5.

## Non-Hermitian position dependent noncommutativity

Given a set operators of ${\hat{X}}, {\hat{Y}}, {\hat{P}}_x, {\hat{P}}_y$ defined on the 2D Hilbert space and satisfy the following commutation relations and all possible permutations of the Jacobi identities^12^

1 $$\begin{aligned}{}[{\hat{X}},{\hat{Y}}]= & {} i\theta (1-\tau {\hat{Y}} +\tau ^2 {\hat{Y}}^2),\quad [{\hat{X}},{\hat{P}}_x ]=i\hbar (1-\tau {\hat{Y}} +\tau ^2 {\hat{Y}}^2),\nonumber \\ {[{\hat{Y}},{\hat{P}}_y ]}= & {} i\hbar (1-\tau {\hat{Y}} +\tau ^2 {\hat{Y}}^2),\quad [{\hat{X}},{\hat{P}}_y]=i\hbar \tau (2\tau {\hat{Y}}{\hat{X}}-{\hat{X}})+ i\theta \tau (2\tau {\hat{Y}}{\hat{P}}_y-{\hat{P}}_y)\nonumber \\ { [{\hat{P}}_x,{\hat{P}}_y]}= & {} 0, \quad \quad \quad \quad \quad \quad \quad \quad \quad {[{\hat{Y}},{\hat{P}}_x]}=0. \end{aligned}$$

where $\theta ,\tau \in (0,1)$ are both deformed parameters that describe the frontier of the Planck scale. The parameter $\tau$ is the GUP deformed parameter^5,48,49^ related to quantum gravitational effects at this scale. The parameter $\theta$ is related to the noncommutativity of the space at this scale^50–53^. In the framework of noncommutative classical or quantum mechanics, this parameter is proportional to the inverse of a constant magnetic field such that $\theta =1/B$^52–54^. Since the algebra (1) describes the space at the Planck scale, then such magnetic fields are necessarily superstrong and may play the role of primordial magnetic fields^55^. Obviously by taking $\tau \rightarrow 0$, we get the $\theta$-deformed space

2 $$\begin{aligned}{}[{\hat{x}}_0,{\hat{y}}_0]= & {} i\theta ,\,\,\,[{\hat{x}}_0,{\hat{p}}_{x_0}]=i\hbar ,\,\,\,[{\hat{y}}_0,{\hat{p}}_{y_0}]=i\hbar ,\nonumber \\ {[{\hat{p}}_{x_0},{\hat{p}}_{y_0}]}= & {} 0,\,\,\quad [{\hat{x}}_0,{\hat{p}}_{y_0}]=0,\,\,\, [{\hat{y}}_0, {\hat{p}}_{x_0}]=0. \end{aligned}$$

Using the asymmetrical Bopp-shift^42^, we can relate the noncommutative operators (2) to the ordinary commutations ones as follows:

3 $$\begin{aligned} {\hat{x}}_0={\hat{x}}_s-\frac{\theta }{2\hbar }{\hat{p}}_{y_s},\quad {\hat{y}}_0={\hat{y}}_s, \end{aligned}$$

where the Hermitian operators ${\hat{x}}_s,{\hat{y}}_s,{\hat{p}}_{x_s},{\hat{p}}_{y_s}$ satisfy the ordinary 2D Heisenberg algebra

4 $$\begin{aligned}{}[{\hat{x}}_s,{\hat{y}}_s]= & {} 0,\,\,\,[{\hat{x}}_s,{\hat{p}}_{x_s}]=i\hbar ,\,\,\,[{\hat{y}}_s,{\hat{p}}_{y_s}]=i\hbar , \nonumber \\ {[}{\hat{p}}_{x_s},{\hat{p}}_{y_s}]= & {} 0,\,\,\, [{\hat{x}}_s,{\hat{p}}_{y_s}]=0,\,\,\,\,\,\,\, [{\hat{y}}_s, {\hat{p}}_{x_s}]=0. \end{aligned}$$

The operators (${\hat{x}}_0, {\hat{y}}_0, {\hat{p}}_{x_0}, {\hat{p}}_{y_0})$ and (${\hat{x}}_s, {\hat{y}}_s, {\hat{p}}_{x_s}, {\hat{p}}_{y_s})$ from the algebras (2) and (4) respectevely, can be interpreted as the set of operators at low energies with the standard representations in position space. However the operators $({\hat{X}}, {\hat{Y}} , {\hat{P}}_x, {\hat{P}}_y)$ of the algebra (1) can be interpreted as the set of operators at high energies with the generalized representation in position space. In terms of the standard flat-Hermitian noncommutative operators (2), we may now represent the algebra (1) as follows

5 $$\begin{aligned} {\hat{X}}=(1-\tau {\hat{y}}_0+\tau ^2{\hat{y}}_0^2){\hat{x}}_0, \quad {\hat{Y}}={\hat{y}}_0,\quad {\hat{P}}_x={\hat{p}}_{x_0},\quad {\hat{P}}_y=(1-\tau {\hat{y}}_0+\tau ^2{\hat{y}}_0^2){\hat{p}}_{y_0}. \end{aligned}$$

Using the asymmetrical Bopp-shift (3), the above relation becomes

6 $$\begin{aligned} {\hat{X}}= & {} (1-\tau {\hat{y}}_s+\tau ^2{\hat{y}}_s^2){\hat{x}}_s-\frac{\theta }{2\hbar }(1-\tau {\hat{y}}_s+\tau ^2{\hat{y}}_s^2){\hat{p}}_{y_s}, \quad {\hat{Y}}={\hat{y}}_s,\nonumber \\ {\hat{P}}_x= & {} {\hat{p}}_{x_s},\quad \quad {\hat{P}}_y=(1-\tau {\hat{y}}_s+\tau ^2{\hat{y}}_s^2){\hat{p}}_{y_s}. \end{aligned}$$

From these representations (5) and (6) follows immediately that some of the operators involved are no longer Hermitian. We observe

7 $$\begin{aligned} {\hat{X}}^\dag ={\hat{X}}-i\theta \tau (1-2\tau {\hat{Y}}),\quad {\hat{Y}}^\dag ={\hat{Y}}, \quad {\hat{P}}_x^\dag = {\hat{P}}_x,\quad {\hat{P}}_y^\dag = {\hat{P}}_y+i\hbar \tau ({\mathbb {I}}-2\tau {\hat{Y}}). \end{aligned}$$

As is apparent, the operators ${\hat{X}}$ and ${\hat{P}}_y$ are not Hermitian. This situation is widely expected in various studies of quantum gravity since at this scale the space-time becomes fuzzy. This fuzziness is a consequence of existence of minimal uncertainties at this scale which induced a loss of Hermicities in *X* and *P* directions. In his elegant paper^56^, Kempf named this type of operators the unsharp degrees of fredoms which describe the space time at short distance. As an immediate consequence of the non Hermicities of the operators *X* and *P* is that, the Hamiltonian of the system involving these operators will in general also not be Hermitian i.e. ${\hat{H}}^\dag ({\hat{X}},{\hat{Y}},{\hat{P}}_x,{\hat{P}}_y)\ne {\hat{H}}({\hat{X}},{\hat{Y}},{\hat{P}}_x,{\hat{P}}_y)$. In order to map these operators into Hermitian ones, some synonymous used concepts are introduced in the literature such as the $\mathcal{PT}\mathcal{}$-symmetry^33–36^, the quasi-Hermiticity^37,38^, the pseudo-Hermiticity^39–41^ or the cryptoHermiticity^42–44^. It has been clarified in^34^ that a non-Hermitian operator ${\mathcal {O}}$ having all eigenvalues real is connected to its Hermitian conjugate ${\mathcal {O}}^\dag$ through a linear, Hermitian, invertible, and bounded metric operator $\rho$ such as $\rho {\mathcal {O}} \rho ^{-1}= {\mathcal {O}}^\dag$. Factorizing this operator into a product of a Dyson operator $\eta$ and its Hermitian conjugate in the form $\rho =\eta ^\dag \eta$, it is established^34^ that the non Hermitian operator can be transformed to an equivalent Hermitian one given by $o=\eta {\mathcal {O}}\eta ^{-1}=o^\dag$. Schematically summarized, the latter can be described by the following sequence of steps

8 $$\begin{aligned} {\mathcal {O}}\ne & {} {\mathcal {O}}^\dag \xrightarrow {\rho } \rho {\mathcal {O}} \rho ^{-1}= {\mathcal {O}}^\dag \xrightarrow {\eta } \eta {\mathcal {O}}\eta ^{-1}=o=o^\dag . \end{aligned}$$

For the case at hand, we find that the Dyson map can be taken to be

9 $$\begin{aligned} \eta =(1-\tau {\hat{Y}} +\tau ^2 {\hat{Y}}^2)^{-1/2}, \end{aligned}$$

so the hidden Hermitian variables ${\hat{x}}, {\hat{y}}, {\hat{p}}_x , {\hat{p}}_y$ can be stated in terms of $\theta$-deformed space operators as follows

10 $$\begin{aligned} {\hat{x}}= & {} \eta {\hat{X}}\eta ^{-1}= (1-\tau {\hat{y}}_0 +\tau ^2 {\hat{y}}_0^2)^{1/2}{\hat{x}}_0 (1-\tau {\hat{y}}_0 +\tau ^2 {\hat{y}}_0^2)^{1/2}={\hat{x}}^\dag , \end{aligned}$$

11 $$\begin{aligned} {\hat{p}}_x= & {} \eta {\hat{P}}_x\eta ^{-1}={\hat{p}}_{x_0}={\hat{p}}_x^\dag , \end{aligned}$$

12 $$\begin{aligned} {\hat{y}}= & {} \eta {\hat{Y}}\eta ^{-1}={\hat{y}}_0=y^\dag , \end{aligned}$$

13 $$\begin{aligned} {\hat{p}}_y= & {} \eta {\hat{P}}_y\eta ^{-1}= (1-\tau {\hat{y}}_0 +\tau ^2 {\hat{y}}_0^2)^{1/2}{\hat{p}}_{y_0} (1-\tau {\hat{y}}_0 +\tau ^2 {\hat{y}}_0^2)^{1/2}={\hat{p}}_y^\dag . \end{aligned}$$

These operators satisfy the same deformed canonical commutation relations as their counterparts in the non-Hermitian version of the theory (1)

14 $$\begin{aligned}{}[{\hat{x}},{\hat{y}}]= & {} i\theta (1-\tau {\hat{y}} +\tau ^2 {\hat{y}}^2),\quad [{\hat{x}},{\hat{p}}_x ]=i\hbar (1-\tau {\hat{y}} +\tau ^2 {\hat{y}}^2),\nonumber \\ {[{\hat{y}},{\hat{p}}_y ]}= & {} i\hbar (1-\tau {\hat{y}} +\tau ^2 {\hat{y}}^2),\quad [{\hat{x}},{\hat{p}}_y]=i\hbar \tau (2\tau {\hat{y}}{\hat{x}}-{\hat{x}})+ i\theta \tau (2\tau {\hat{y}}{\hat{p}}_y-{\hat{p}}_y),\nonumber \\ { [{\hat{p}}_x,{\hat{p}}_y]}= & {} 0, \quad \quad \quad \quad \quad \quad \quad \quad {[{\hat{y}}, {\hat{p}}_x]}=0. \end{aligned}$$

As is well established in^34^, a consequence of the non-Hermiticity of an operator ${\mathcal {O}}$, its eigenstates no longer form an orthonormal basis and the Hilbert space representation has to be modified. This is achieved by utilizing the operator $\rho$ as a metric to define a new inner product $\langle . \,|\, .\rangle _\rho$ in terms of the standard inner product $\langle .\, |\,.\rangle$ defined as

15 $$\begin{aligned} \langle \Phi | \Psi \rangle _\rho :=\langle \Phi |\rho \Psi \rangle , \end{aligned}$$

for arbitrary states $\langle \Phi |$ and $| \Psi \rangle$. The observables ${\mathcal {O}}$ are then Hermitian with respect to this new metric

16 $$\begin{aligned} \langle \Phi |{\mathcal {O}} \Psi \rangle _\rho =\langle {\mathcal {O}}\Phi |\rho \Psi \rangle . \end{aligned}$$

An important physical consequence resulting from the algebra (1), is the loss of the Hermicity of certain operators which deformed the structure of the Hilbert space (15) as were predicted by the theory of Kempf *et al*^5^. In the next section, let us study Heisenberg’s uncertainty principle applied to a simultaneous measurement of operators of this algebra.

## Minimal and maximal uncertainty measurements

For the system of operators satisfying the commutation relations in (1), the generalized uncertainty principle is defined as follows

17 $$\begin{aligned} \Delta A\Delta B\ge \frac{1}{2}|\langle [{\hat{A}},{\hat{B}}]\rangle _\rho |\quad \text{ for } \quad {\hat{A}},{\hat{B}}\in \{{\hat{X}},{\hat{Y}}, {\hat{P}}_x,{\hat{P}}_y\}, \end{aligned}$$

where $\Delta A=\sqrt{\langle ({\hat{A}}-\langle {\hat{A}}\rangle _\rho )^2\rangle _\rho }$ and for ${\hat{B}}$. An interesting features can be observed through the following uncertainty relations:

18 $$\begin{aligned} \Delta X\Delta Y\ge & {} \frac{\theta }{2}\left( 1-\tau \langle {\hat{Y}}\rangle _\rho +\tau ^2\langle {\hat{Y}}^2\rangle _\rho \right) , \end{aligned}$$

19 $$\begin{aligned} \Delta Y\Delta P_y\ge & {} \frac{\hbar }{2}\left( 1-\tau \langle {\hat{Y}}\rangle _\rho +\tau ^2\langle {\hat{Y}}^2\rangle _\rho \right) . \end{aligned}$$

For $\tau =0$, we recover the uncertainty relations of ordinary noncommutative quantum mechanics

20 $$\begin{aligned} \Delta x_0\Delta y_0\ge & {} \frac{\theta }{2}, \end{aligned}$$

21 $$\begin{aligned} \Delta y_s\Delta p_{y_s}\ge & {} \frac{\hbar }{2}. \end{aligned}$$

**Figure 1:**
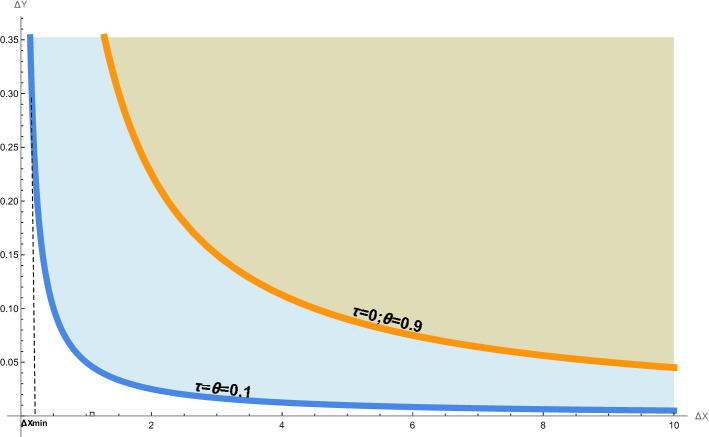
The quantum uncertainty relation (20) and the quantum gravitational modification (22). The quantum uncertainty (orange curve) is plotted together with a modified gravitaional uncertainty relation (blue curve) respectively. The bue shaded region represents states that are allowed by quantum gravity but forbidden by regular quantum mechanics. The brown shade region represents states allowed by both quantum gravity and quantum mechanics.

In the Fig. 1, we plot the modified gravitational uncertainty (18) together with the ordinary quantum uncertainty (20). Alternatively, the generalized Heisenberg uncertainty (19) and the ordinary one (21) are plotted in the Fig. 4. As we pointed out in the above section, the uncertainty relations (20) and (21) describe states of representations at low energies characterized by a large value of the noncommutative parameter, e.g. $\theta =0.9$. However, the generalized uncertainties (18) and (19) describes the high energy characterized by absolute minimal values of parameters $\theta$ and $\tau$ ( e.g. $\theta =\tau =0.1$) which describe the Planck scale.

*i*) For the uncertainty relation (18), using $\langle {\hat{Y}}^2\rangle _\rho =\Delta Y^2+\langle {\hat{Y}}\rangle _\rho ^2$, the inequality (18) becomes

22 $$\begin{aligned} \Delta X\Delta Y\ge & {} \frac{\theta }{2}\left( 1 -\tau \langle {\hat{Y}}\rangle _\rho +\tau ^2\langle {\hat{Y}}\rangle _\rho ^2+\tau ^2 \Delta Y^2\right) . \end{aligned}$$

This Eq. (22) can be rewritten as a second order equation of $\Delta Y$

23 $$\begin{aligned} \Delta Y^2- \frac{2}{\tau \theta }\Delta X \Delta Y +\langle {\hat{Y}}\rangle _\rho \left( \langle {\hat{Y}}\rangle _\rho -\frac{1}{\tau }\right) +\frac{1}{\tau ^2}\le 0. \end{aligned}$$

By setting the Eq. (24) into

24 $$\begin{aligned} \Delta Y^2- \frac{2}{\tau \theta }\Delta X \Delta Y +\langle {\hat{Y}}\rangle _\rho \left( \langle {\hat{Y}}\rangle _\rho -\frac{1}{\tau }\right) +\frac{1}{\tau ^2}=0, \end{aligned}$$

the solution are given by

25 $$\begin{aligned} \Delta Y=\frac{\Delta X}{\theta \tau ^2}\pm \sqrt{\left( \frac{\Delta X}{\theta \tau ^2}\right) ^2 -\frac{\langle {\hat{Y}}\rangle }{\tau }\left( \tau \langle {\hat{Y}}\rangle -1\right) -\frac{1}{\tau ^2}}. \end{aligned}$$

The existence of real absolute minimal solution $\Delta X_{min}$ of Eq. (25) is obtained by fixing $\langle {\hat{Y}}\rangle =0$ in *X*-direction

26 $$\begin{aligned} \Delta X_{min}=\tau \theta =\frac{\tau }{B}=l_{min}. \end{aligned}$$

The Eq. (26) clearly shows that the absolute maximal length $\Delta Y_{max}$ corresponding to $\Delta X_{min}$ is given by

27 $$\begin{aligned} \Delta Y_{max}=\frac{1}{\tau }=l_{max}. \end{aligned}$$

**Figure 2:**
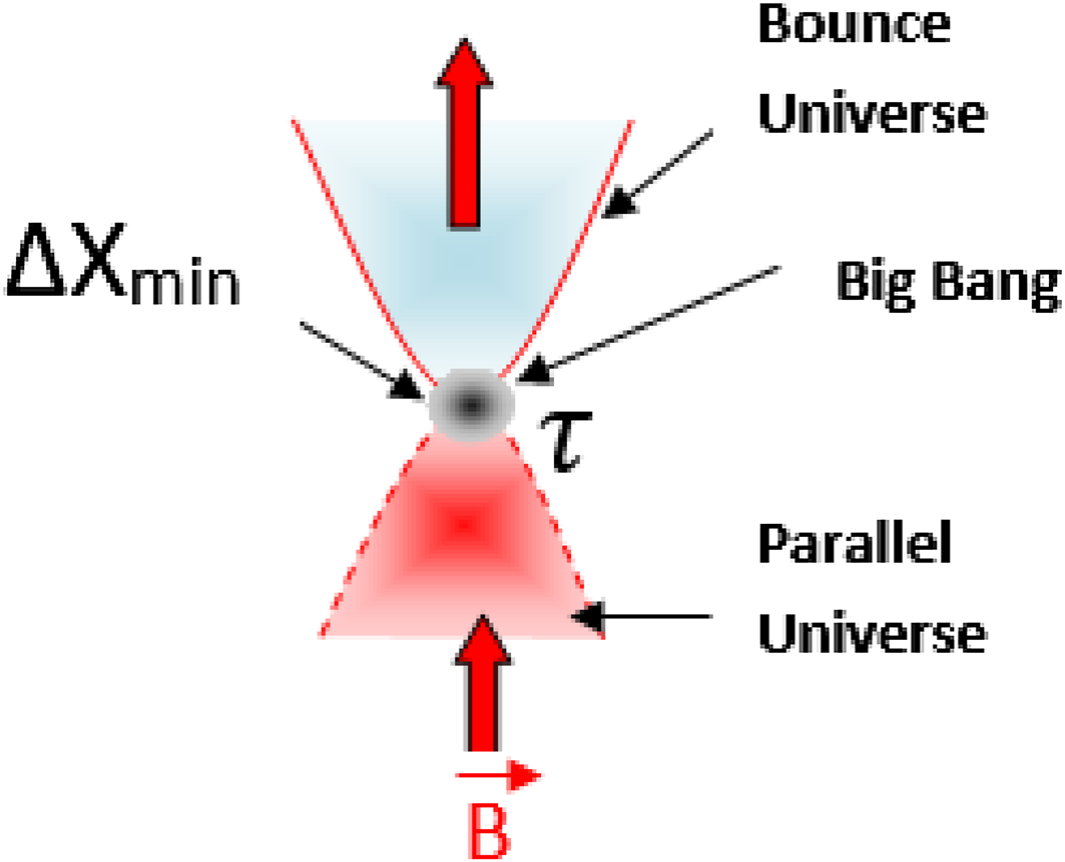
Representation of minimal length scale $\Delta X_{min}$.

Both solutions $\Delta X_{min}$ and $\Delta Y_{max}$ confirm Perivolaropoulos’s prediction^11^. Different versions of minimal length uncertainties have been introduced in the literature^56–65^ which significantly improve the one proposed by Kempf et al^5^. These minimal length uncertainties are widely known to induce a singularity of position representation at the Planck scale i.e., they are inevitably bounded by minimal quantities beyond which any further localization of particles is not possible. In contrast to these findings, the obtained minimal length $\Delta X_{min}=\tau /B$ induces a broken singularity at the Planck scale as a result of an external magnetic field *B*. In fact this scenario can be regarded as the Landau quantization problem^66^, in which the Planck scale which is limited by the weak quantum gravitational field $\tau$, is orthogonally subjected to the parallel universe superstrong magnetic field, causing it to bounce at this minimal point. This broken singularity manifested by a big bang unifies the weak quantum gravitational field and the superstrong magnetic field as minimal length (see Fig. 2). A simultaneous measurement of the minimal length $\Delta X_{min}$ and the maximal length $\Delta Y_{max}$ generates the inverse of the magnetic field as follows

28 $$\begin{aligned} \Delta X_{min} \Delta Y_{max}=\frac{1}{B}=l_{min}l_{max}. \end{aligned}$$

If we iterate the process (28) *n* times, we generate a sequence of minimal lengths alternated by maximal lengths, such as

29 $$\begin{aligned} ...l_{min}l_{max}l_{min}l_{max}...\simeq \frac{1}{B^{n}}. \end{aligned}$$

**Figure 3:**
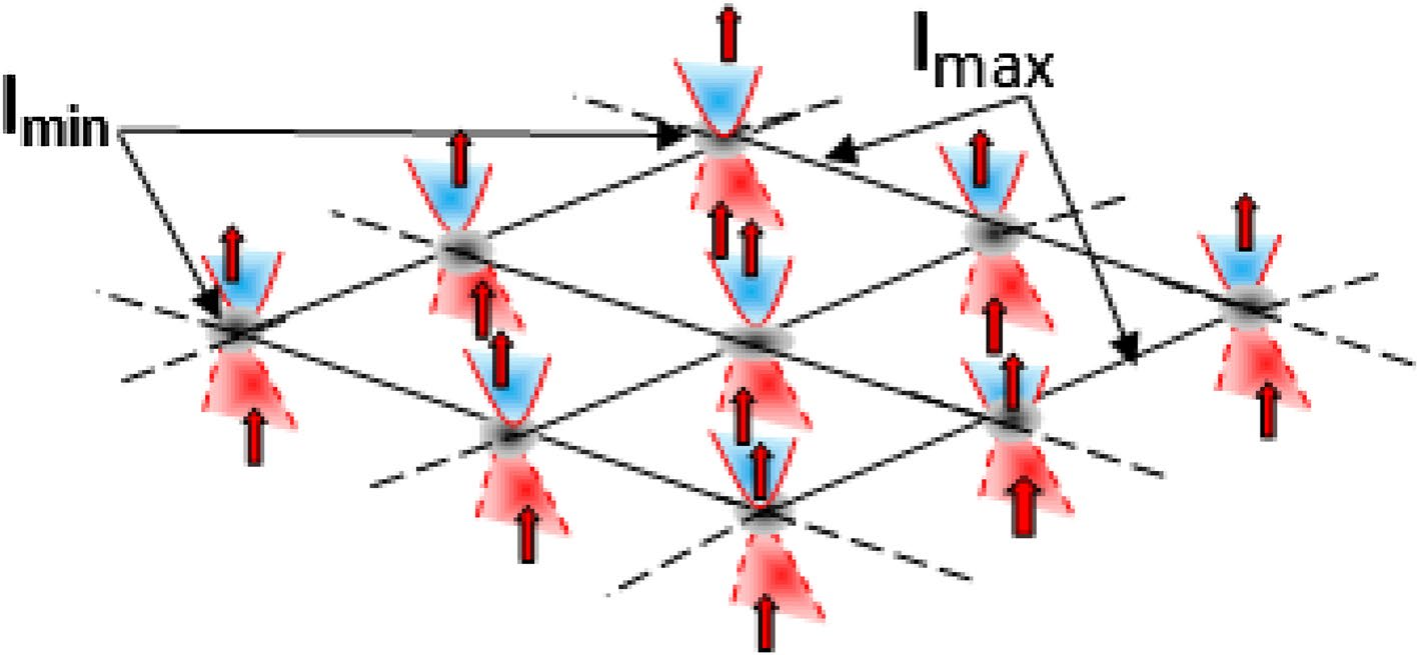
A simultaneous representation of minimal lengths $\Delta X_{min}$ and maximal lengths $\Delta X_{max}$.

This sequence resembles a lattice structure in which each site represented by $l_{min}$ is separated by $l_{max}$ (see Fig. 3).

ii) Repeating the same calculation and argumentation in the situation of uncertainty relation (27) for simultaneous ${\hat{Y}},{\hat{P}}_y$-measurement, we find the absolute maximal uncertainty $\Delta Y_{max}$ (27) and an absolute minimal uncertainty momentum $\Delta P_{y_{min}}$ for $\langle {\hat{Y}}\rangle _\rho =0$

30 $$\begin{aligned} \Delta Y_{max}=\frac{1}{\tau }=l_{max},\quad \Delta P_{y_{min}}=\hbar \tau =p_{min}. \end{aligned}$$

**Figure 4:**
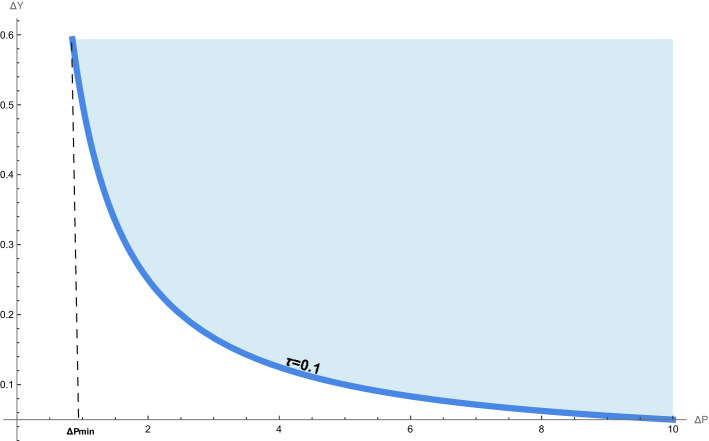
Generalized Heisenberg uncertainty (19) in accordance with ordinary Heisenberg uncertaity (21) after rescaling to dimensionless form. The blue shade region represents states allowed by both quantum gravity and quantum mechanics. This result is consistent with that of Perivolaropoulos^11^.

These results (30) are consistent with the ones obtained by Perivolaropoulos^9^. From these results, It’s worth noting that the GUP is reduced into $\Delta Y_{max}\Delta P_{y_{min}}=\hbar$. It is well known from the Heisenberg principle that the latter relation can be cast into

31 $$\begin{aligned} \Delta Y_{max}\Delta E=\hbar c\implies \Delta E=\frac{\hbar c }{\Delta Y_{max}}, \end{aligned}$$

where $\Delta E= \Delta P_{y_{min}} c$. Unlike the results obtained in the minimal length scenarios^5,8,57–64^, here the required uncertainty energy is weak since the dimension of length $\Delta Y_{max}$ is very large. This indicates that a maximal localization of quantum gravity induces weak energies for its measurement. Let us now consider the equation $\Delta Y_{max} =\Delta t c$. Inserting this equation in (31), one obtains

32 $$\begin{aligned} \Delta t=\frac{\hbar }{\Delta E}. \end{aligned}$$

Since, the uncertainty energy is low in this space due to the maximal measurement of quantum gravity, then its time $\Delta t$ strongly increases i.e., the time runs more slowly in this space. In contrast to minimal length theories, the concept of maximal length quantum gravity developed in this paper admits a close analogy with the properties of gravity in GR in the sense that the gravitational field becomes stronger for heavy systems that curve the space, allowing the surrounding light systems to fall down with low energies. The resulting time inside of this space is dilated and length contraction takes effect. As will be demonstrated in the next section, the increase of the quantum gravitational parameter $\tau$ in an infinite square well potential curves the quantum levels to enable enable the particles to jump from one state to another with low energy and with high probability densities. The wavefunction compresses and contracts inward as one increases the effect of quantum gravitational effects.

iii) Finally, simultaneous measurements of operators $({\hat{X}}, {\hat{P}}_y)$, $({\hat{X}}, {\hat{P}}_x)$ and $({\hat{X}}, {\hat{P}}_y)$ are spatial isotropy since there is no minimal/maximal length or minimal momentum in their measurements.

Moreover, by repeating the GUP calculations with the hidden position-dependent noncommutativity (14), one generates the same uncertainty measurements

33 $$\begin{aligned} \Delta x_{min}=\theta \tau ,\quad \Delta y_{max}=\frac{1}{\tau },\quad \Delta p_{min}=\hbar \tau . \end{aligned}$$

**Figure 5:**
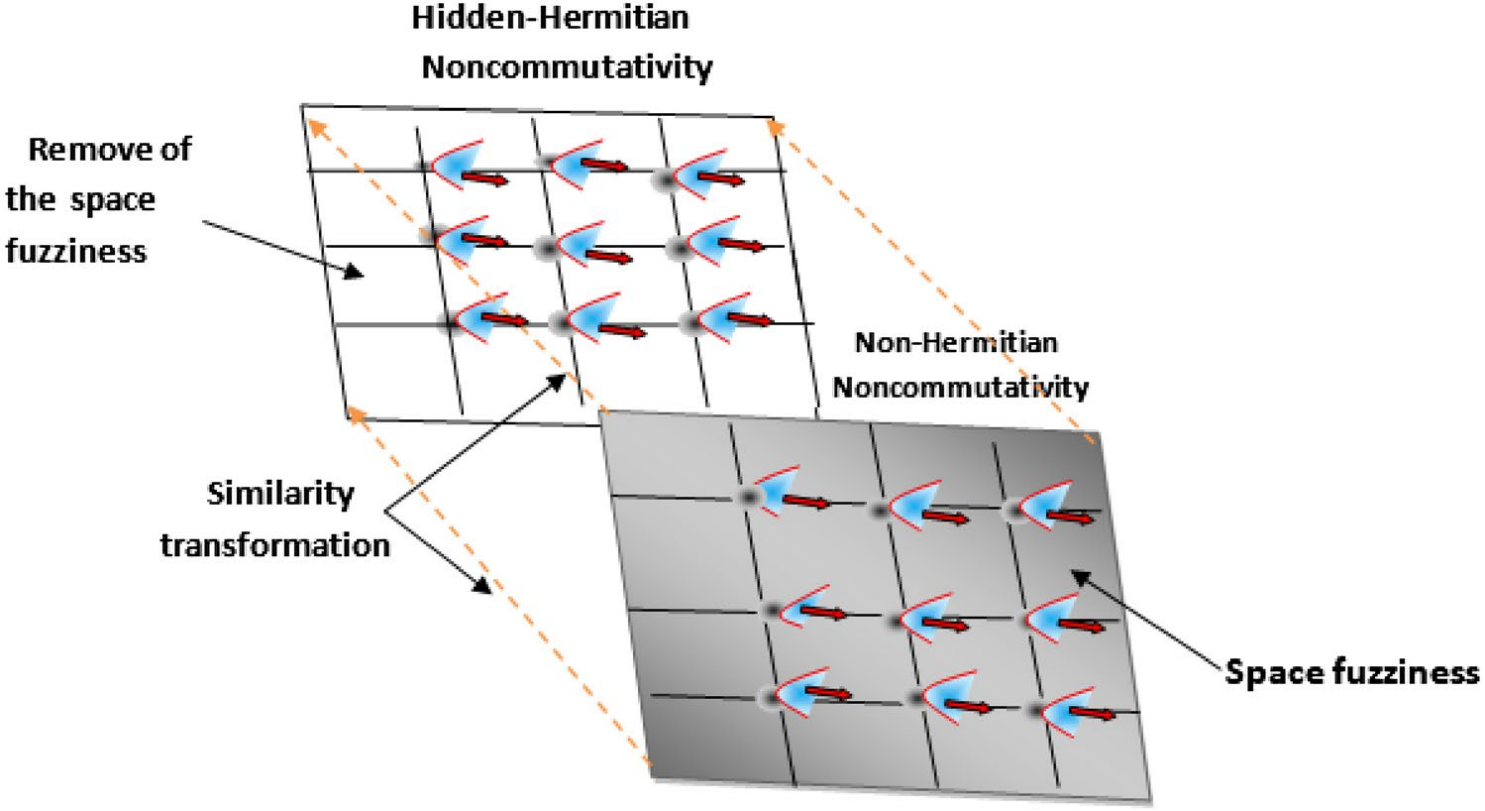
Hidden Hermitian noncommutativity and Non-Hermitian noncommutativity.

This indicates that the Dyson map does not remove the characteristics of quantum gravity at this scale i.e., it only removes the fuzziness induced by the singular points by shedding light on the hidden Hermitian space. Consequently, particles can be localized in precise ways in this new space. This situation could be compared to the gravitational holographic principle where the real information inside the black hole is virtually projected onto its event horizon. Taking this into consideration, the simultaneous representation of minimal length $\Delta X_{min}$ and maximal length $\Delta Y_{max}$ (Fig. 3) can be illustrated as follows (Fig. 5)

##  Hidden-Hermitian free particle Hamiltonian in a box

The Hamiltonian of free particle in 2D non-Hermitian position-dependent noncommutative space reads as follows

34 $$\begin{aligned} {\hat{H}}_F=\frac{1}{2m_0}\left( {\hat{P}}_x^2+{\hat{P}}_y^2\right) . \end{aligned}$$

As mentioned, any Hamiltonian depending on the operators ${\hat{X}}$ or ${\hat{P}}_y$ will obviously no longer be Hermitian. Thus, using the relations (5), we can transform the Hamiltonian (34) into the standard $\theta$-deformed operator (2) as follows

35 $$\begin{aligned} {\hat{H}}_F=\frac{1}{2m_0}\left[ {\hat{p}}_{x_0}^2+\left( 1-\tau {\hat{y}}_0+\tau ^2 {\hat{y}}_0^2\right) ^2 {\hat{p}}_{y_0}^2-i\hbar \tau (1+2\tau {\hat{y}}_0)(1-\tau {\hat{y}}_0 +\tau ^2{\hat{y}}_0^2){\hat{p}}_{y_0}\right] . \end{aligned}$$

Evidently this Hamiltonian is non-Hermitian ${\hat{H}}\ne {\hat{H}}^\dag$. Thus, the Hermicity requirement of this operator is achieved by means of a similarity transformation using the Dyson map. Thus, the Hermitian counterpart Hamiltonian becomes

36 $$\begin{aligned} {\hat{h}}_F=\eta {\hat{H}}_F\eta ^{-1}= \frac{1}{2m_0}\left( {\hat{p}}_x^2+{\hat{p}}_y^2\right) . \end{aligned}$$

Using the relation (11,13), we rewrite the Hamiltonian in terms of the $\theta$-noncommutative operators

37 $$\begin{aligned} {\hat{h}}_F=\frac{1}{2m_0}\left[ {\hat{p}}_{x_0}^2+ (1-\tau {\hat{y}}_0 +\tau ^2 {\hat{y}}_0^2)^{1/2}{\hat{p}}_{y_0} (1-\tau {\hat{y}}_0 +\tau ^2 {\hat{y}}_0^2) {\hat{p}}_{y_0} (1-\tau {\hat{y}}_0 +\tau ^2 {\hat{y}}_0^2)^{1/2}\right] . \end{aligned}$$

Appealing to the nonsymmetric Bopp-shift (3), we may rewrite the above Hamiltonian as follows

38 $$\begin{aligned} {\hat{h}}_F=\frac{1}{2m_0}\left[ {\hat{p}}_{x_s}^2+ (1-\tau {\hat{y}}_s +\tau ^2 {\hat{y}}_s^2)^{1/2}{\hat{p}}_{y_s} (1-\tau {\hat{y}}_s +\tau ^2 {\hat{y}}_s^2) {\hat{p}}_{y_s} (1-\tau {\hat{y}}_s +\tau ^2 {\hat{y}}_s^2)^{1/2}\right] . \end{aligned}$$

The time-independent Schrödinger equation is given by

39 $$\begin{aligned} {\hat{h}}_F\psi (x_s,y_s)= & {} E\psi (x_s,y_s),\nonumber \\ ({\hat{h}}_F^x+{\hat{h}}_F^y) \psi (x_s,y_s)= & {} E\psi (x_s,y_s). \end{aligned}$$

As it is clearly seen, the system is decoupled and the solution to the eigenvalue Eq. (39) is given by

40 $$\begin{aligned} \psi (x_s,y_s)= \psi (x_s)\psi (y_s), \quad E=E_x+E_y \end{aligned}$$

where $\psi (x_s)$ is the wave function in the $x_s$-direction and $\psi (y_s)$ the wave function in the $y_s$-direction. Since the particle is free in the $x_s$-direction, the wave function is given by^26^

41 $$\begin{aligned} \psi _k(x_s)=\int _{-\infty }^{+\infty } dk g(k)e^{ikx_s}, \end{aligned}$$

where *g*(*k*) determines the shape of the wave packet and the energy spectrum is continuous^26^

42 $$\begin{aligned} E_x=E_k=\frac{\hbar ^2 k^2}{2m_0}. \end{aligned}$$

In $y_s$-direction, we have to solve the following equation

43 $$\begin{aligned} \frac{1}{2m_0} (1-\tau {\hat{y}}_s +\tau ^2 {\hat{y}}_s^2)^{1/2}{\hat{p}}_{y_s} (1-\tau {\hat{y}}_s +\tau ^2 {\hat{y}}_s^2) {\hat{p}}_{y_s} (1-\tau {\hat{y}}_s +\tau ^2 {\hat{y}}_s^2)^{1/2}\psi (y_s)=E_y\psi (y_s). \end{aligned}$$

**Figure 6:**
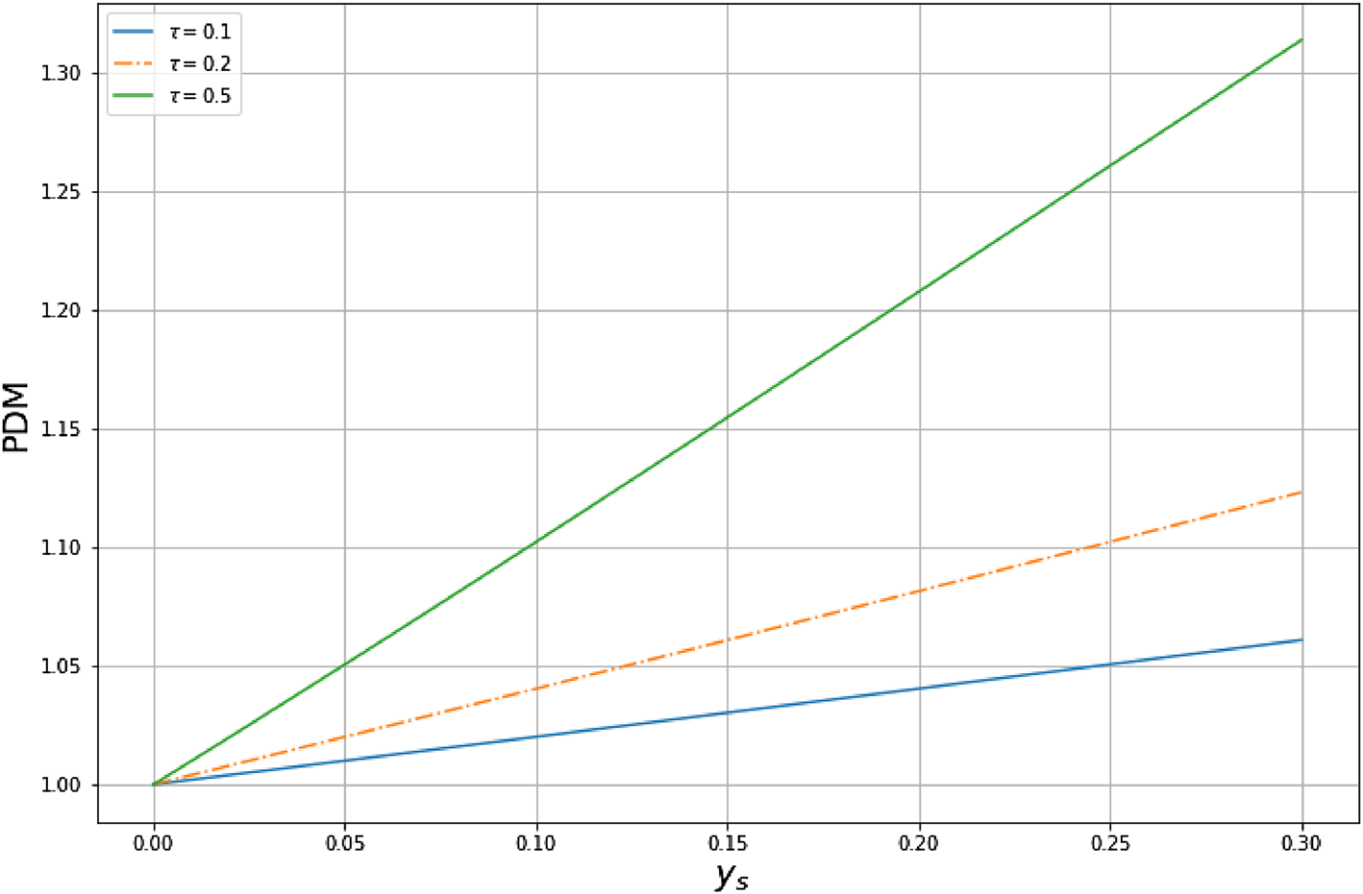
PDM versus the position $y_s$ for different values of $\tau$.

This equation is an agreement with the one introduced by von Roos^67^ for systems with a position-dependent mass (PDM) operator and it can be rewritten as^68^

44 $$\begin{aligned} \left( -\frac{\hbar ^2}{2m_0}\root 4\, \of {\frac{m_0}{m(y_s)}}\frac{\partial }{\partial _{y_s}}\sqrt{\frac{m_0}{m(y_s)}}\frac{\partial }{\partial _{y_s}}\root 4\, \of {\frac{m_0}{m(y_s)}}\right) \psi (y_s)=E_y \psi (y_s), \end{aligned}$$

where

45 $$\begin{aligned} m({\hat{y}}_s)=\frac{m_0}{(1-\tau {\hat{y}}_s +\tau ^2 {\hat{y}}_s^2)^2 }, \end{aligned}$$

being the PDM of the system strongly pertubated by quantum gravity^13^. The PDM is illustated in Fig. 6 as a function of the position $y_s$ $(0< y_s < 0.3)$. In this description, the effective mass of $m({\hat{y}}_s)$ increases with $\tau$. This indicates that quantum gravitational fields increase with $m({\hat{y}}_s)$. Otherwise, by increasing experimentally the PDM, one can make the quantum gravitational effects stronger for a measurement through the variation of the PDM of the system. Furthermore, the increase of PDM with the quantum gravitational effect will be a consequence of the deformation of the quantum energy levels, allowing the particle to jump from one state to another with low energy Figure 8. This observation is perfectly analogous to the theory of GR in which massive objects induce strong gravitational fields and curve space, allowing the surrounding light systems to fall down with low energies..

The Eq. (44) can be conveniently rewritten by means of the transformation $\psi (y_s)=\root 4\, \of {m(y_s)/m_0}\phi (y_s)$ as in^68^

46 $$\begin{aligned} -\frac{\hbar ^2}{2m_0}\left( \sqrt{\frac{m_0}{m(y_s)}}\frac{\partial }{\partial _{y_s}}\right) ^2\phi (y_s)=E_y \phi (y_s),\quad \text{ with } \quad E_y >0, \end{aligned}$$

or

47 $$\begin{aligned} -\frac{\hbar ^2}{2m_0}\left[ (1-\tau y_s+\tau ^2 y_s^2)^2\frac{\partial ^2}{\partial _{y_s^2}}- \tau (1-2\tau y_s)(1-\tau y_s+\tau ^2 y_s^2)\frac{\partial }{\partial _{y_s}}\right] \phi (y_s)=E_y \phi (y_s). \end{aligned}$$

The solution of this Eq. (47) is given by^13^

48 $$\begin{aligned} \phi _\lambda (y_s)= & {} A\exp \left( i\frac{2\lambda }{\tau \sqrt{3}} \left[ \arctan \left( \frac{2\tau y_s-1}{\sqrt{3}}\right) +\frac{\pi }{6}\right] \right) , \end{aligned}$$

49 $$\begin{aligned} \psi _\lambda (y_s)= & {} \frac{A}{\sqrt{1-\tau y_s+\tau ^2 y_s^2}}\exp \left( i\frac{2\lambda }{\tau \sqrt{3}} \left[ \arctan \left( \frac{2\tau y_s-1}{\sqrt{3}}\right) +\frac{\pi }{6}\right] \right) , \end{aligned}$$

where $\lambda =\frac{\sqrt{2m_0E_y}}{\hbar }$ and A is the normalization constant. We notice that if the standard wave-function $\psi _\lambda (y_s)$ is normalized, then $\phi _\lambda (y_s)$ is normalized under a $\tau$-deformed integral. Indeed, we have

50 $$\begin{aligned} \int _{-\infty }^{+\infty } dy_s\psi _\lambda ^*(y_s)\psi _\lambda (y_s)= \int _{-\infty }^{+\infty }\frac{dy_s}{1-\tau y_s+\tau ^2 y_s^2} \phi _\lambda ^*(y_s)\phi _\lambda (y_s)=1. \end{aligned}$$

Based on this Eq. (50), the normalized constant *A* is determined as follows

51 $$\begin{aligned} 1= & {} \int _{-\infty }^{+\infty }\frac{dy_s}{1-\tau y_s+\tau ^2 y_s^2} \phi _\lambda ^*(y_s)\phi _\lambda (y_s) \end{aligned}$$

52 $$\begin{aligned}= & {} A^2 \int _{-\infty }^{+\infty }\frac{dy_s}{1-\tau y_s+\tau ^2 y_s^2}, \end{aligned}$$

so, we find

53 $$\begin{aligned} A=\sqrt{\frac{\tau \sqrt{3}}{2\pi }}. \end{aligned}$$

The next important point concerns, is the quantization of the energy spectrum; we will show below that this property comes directly from the orthogonality of these solutions. Since the operator ${\hat{h}}_y$ is Hermitian, then the corresponding eigenfunctions $\psi _\lambda (y_s)$ are orthogonal. This property can be shown by considering the integral

54 $$\begin{aligned} \int _{-\infty }^{+\infty } dy_s{\hat{h}}_y^\dag \psi _{\lambda '}^*(y_s)\psi _{\lambda }(y_s)= \int _{-\infty }^{+\infty } dy_s{\hat{\psi }}_\lambda ^*(y_s){\hat{h}}_y\psi _\lambda (y_s), \end{aligned}$$

which becomes, after an integration by parts,

55 $$\begin{aligned} E_{\lambda '}\int _{-\infty }^{+\infty } dy_s \psi _{\lambda '}^*(y_s)\psi _{\lambda }(y_s)= E_{\lambda } \int _{-\infty }^{+\infty } dy_s\hat{\psi }_{\lambda '}^*(y_s)\psi _\lambda (y_s). \end{aligned}$$

Since these two integrals are equal, one has

56 $$\begin{aligned} (E_{\lambda '}-E_{\lambda })\int _{-\infty }^{+\infty } dy_s \psi _{\lambda '}^*(y_s)\psi _{\lambda }(y_s)= & {} 0, \end{aligned}$$

57 $$\begin{aligned} (E_{\lambda '}-E_{\lambda })\int _{-\infty }^{+\infty } dy_s \frac{A^2}{1-\tau y_s+\tau ^2 y_s^2}e^{i\frac{2(\lambda -\lambda ')}{\tau \sqrt{3}} \left[ \arctan \left( \frac{2\tau y_s-1}{\sqrt{3}}\right) +\frac{\pi }{6}\right] }= & {} 0, \end{aligned}$$

58 $$\begin{aligned} \sin \left( \frac{\lambda -\lambda '}{\tau \sqrt{3}}\pi \right)= & {} 0. \end{aligned}$$

The quantization follows from the Eq. (58) and leads to the equation

59 $$\begin{aligned} \frac{\lambda -\lambda '}{\tau \sqrt{3}}\pi= & {} n\pi \nonumber \\ \lambda -\lambda '= & {} \lambda _n=\tau n\sqrt{3}, \quad \quad \quad n\in {\mathbb {N}}, \end{aligned}$$

where one notices the case $n=0$ i.e. $\lambda =\lambda '$, corresponding to the normalization condition considered in (54). Then, the energy spectrum of the particle is written as

60 $$\begin{aligned} E_y=E_n=\frac{3\tau ^2 \hbar ^2}{2m_0}n^2. \end{aligned}$$

As it is clairly obtained, the presence of this deformed parameter $\tau$ in $y_s$-direction quantized the energy of a free particle. This fact comes to confirm the fundamental property of gravity which consists of contracting and discretizing the matter.

Then, the total eigensystem is given by

61 $$\begin{aligned} E={\left\{ \begin{array}{ll} E_x= \frac{\hbar ^2 k^2}{2m_0},\\ E_y=\frac{3\tau ^2 \hbar ^2}{2m_0}n^2. \end{array}\right. } \end{aligned}$$

and

62 $$\begin{aligned} \psi (x_s,y_s)={\left\{ \begin{array}{ll} \psi _k(x_s)= \int _{-\infty }^{+\infty } dk g(k)e^{ikx_s}, \\ \psi _n(y_s)= \frac{A}{\sqrt{1-\tau y_s+\tau ^2 y_s^2}}\exp \left( i 2n \left[ \arctan \left( \frac{2\tau y_s-1}{\sqrt{3}}\right) +\frac{\pi }{6}\right] \right) . \end{array}\right. } \end{aligned}$$

Now, we consider the above free particle of mass $m_0$ captured in a two-dimensional box of length $0\le x_s\le a$ and heigth $0\le y_s\le a$. The boundaries of the box are located. We impose the wave functions $\psi (0)=0=\psi (a)$. The eigensystems in $x_s$-direction are given by

63 $$\begin{aligned} \psi _n(x_s)=\sqrt{\frac{2}{a}}\sin \left( \frac{n\pi }{a}x_s\right) , \quad E_n=n^2\frac{\pi ^2\hbar ^2}{2m_0a^2},\quad E_1=\frac{\pi ^2\hbar ^2}{2m_0a^2}. \end{aligned}$$

Taking the results (63) as a witness, we study what follows the influence of the deformed parameter $\tau$ on the system. In $y_s$-direction, the solution is given by

64 $$\begin{aligned} \psi _k(y_s)=\frac{B}{\sqrt{1-\tau y_s+\tau ^2 y_s^2}}\exp \left( i\frac{2k}{\tau \sqrt{3}} \left[ \arctan \left( \frac{2\tau y_s-1}{\sqrt{3}}\right) +\frac{\pi }{6}\right] \right) , \end{aligned}$$

where $k=\frac{\sqrt{2m_0E'}}{\hbar }$. Then by normalization, $\langle \psi _k|\psi _k\rangle =1$, we have

65 $$\begin{aligned} 1= & {} B^2\int _{0}^{a}\frac{dy_s}{1-\tau y_s+\tau ^2 y_s^2}, \end{aligned}$$

so we find

66 $$\begin{aligned} B=\sqrt{\frac{\tau \sqrt{3}}{2}} \left[ \arctan \left( \frac{2\tau a-1}{\sqrt{3}}\right) +\frac{\pi }{6}\right] ^{-1/2}. \end{aligned}$$

Based on the reference^8^, the scalar product of the formal eigenstates is given by

67 $$\begin{aligned} \langle \psi _{k'}|\psi _k\rangle= & {} \frac{\tau \sqrt{3}}{2(k-k')\left[ \arctan \left( \frac{2\tau a-1}{\sqrt{3}}\right) \right] }\sin \left( \frac{2(k-k')\left[ \arctan \left( \frac{2\tau a-1}{\sqrt{3}}\right) \right] }{\tau \sqrt{3}}\right) . \end{aligned}$$

This relation shows that, the normalized eigenstates (64) are no longer orthogonal. However, if one tends $(k-k')\rightarrow \infty$, these states become orthogonal

68 $$\begin{aligned} \lim _{(k-k')\rightarrow \infty } \langle \psi _{k'}|\psi _{k}\rangle =0. \end{aligned}$$

**Figure 7:**
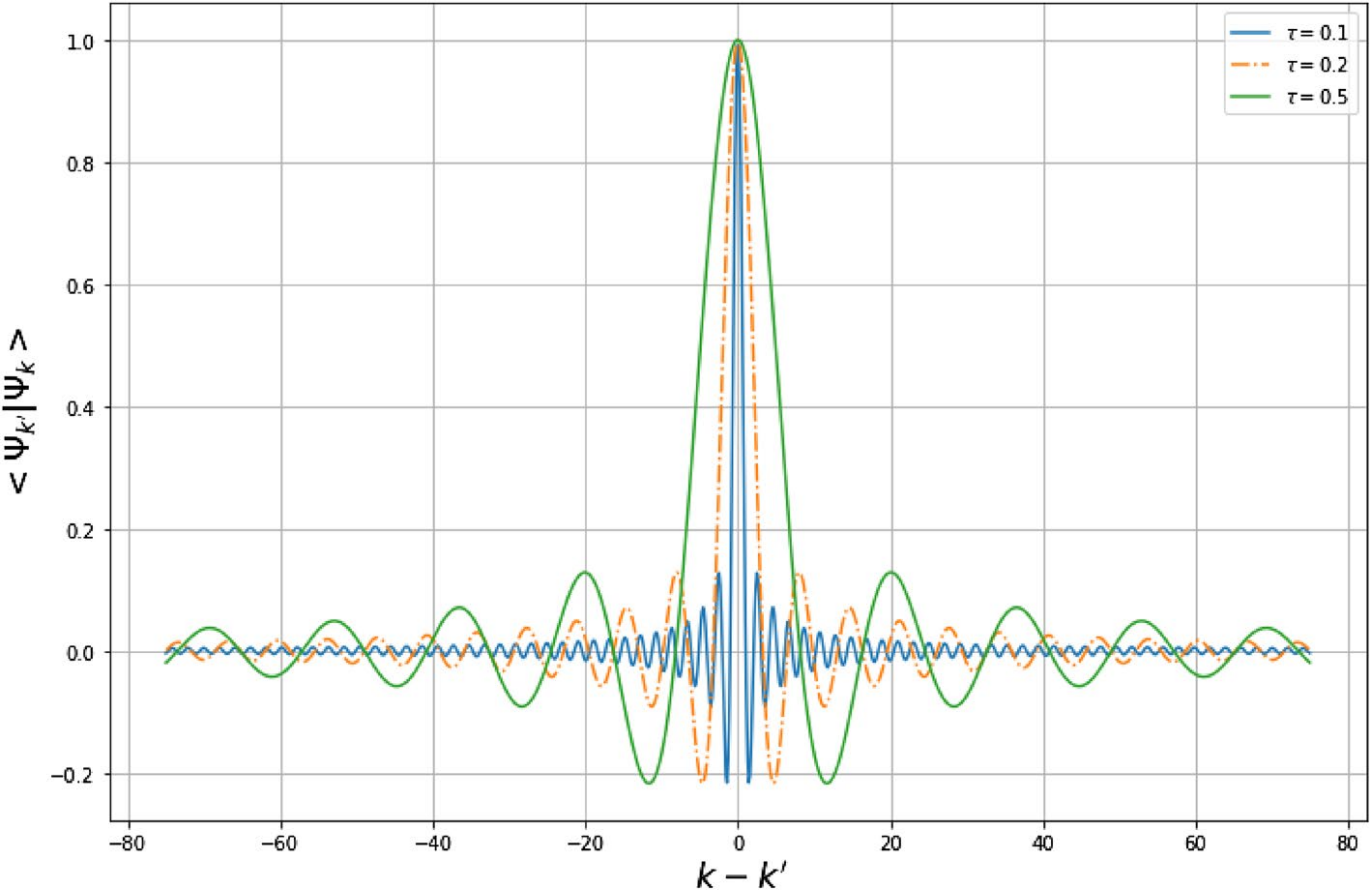
Variation of $\langle \psi _{k'}|\psi _k\rangle$ versus $k-k'$ with $a=1$.

These properties show that, the states $|\psi _k\rangle$ are essentially Gaussians centered at $(k-k')\rightarrow 0$ (see Fig. 7). This observation indicates primordial fluctuations at this scale and these fluctuations increase with the quantum gravitational effects. The states $|\psi _k\rangle$ can be compared to the coherent states of harmonic oscillators^69–72^ which are known as states that mediate a smooth transition between the quantum and classical worlds. This transition is manifested by the saturation of the Heisenberg uncertainty principle $\Delta _z q\Delta _z p=\hbar /2$. In comparison with coherent states of harmonic oscillator, the states $|\psi _{k}\rangle$ strongly saturate the GUP ($\Delta _{\psi _{k}} X\Delta _{\psi _{k}}P=\hbar$) at the Planck scale and could be used to describe the transition states between the quantum world and unknown world for which the physical descriptions are out of reach.

We suppose that, the wave function satisfies the Dirichlet condition i.e., it vanishes at the boundaries $\psi _k(0)=0=\psi _k(a)$. Thus, using especially the boundary condition $\psi _k(0)=0$, the above wavefunctions (64) becomes

69 $$\begin{aligned} \psi _k (y_s) = \frac{B}{\sqrt{1-\tau y_s+\tau ^2 y_s^2}}\sin \left( \frac{2k}{\tau \sqrt{3}}\left[ \arctan \left( \frac{2\tau y_s-1}{\sqrt{3}}\right) +\frac{\pi }{6}\right] \right) . \end{aligned}$$

The quantization follows from the boundary condition $\psi _k(a)=0$ and leads to the equation

70 $$\begin{aligned} \frac{2k_n}{\tau \sqrt{3}}\left[ \arctan \left( \frac{2\tau a-1}{\sqrt{3}}\right) +\frac{\pi }{6}\right]= & {} n\pi \quad \,\,\text{ with }\quad n\in {\mathbb {N}}^*, \end{aligned}$$

71 $$\begin{aligned} k_n= & {} \frac{\pi \tau \sqrt{3}n}{2\left[ \arctan \left( \frac{2\tau a-1}{\sqrt{3}}\right) +\frac{\pi }{6}\right] }. \end{aligned}$$

Then, the energy spectrum of the particle is written as

72 $$\begin{aligned} E_n'= & {} \frac{3\pi ^2 \tau ^2\hbar ^2n^2}{8m_0 \left[ \arctan \left( \frac{2\tau a-1}{\sqrt{3}}\right) +\frac{\pi }{6}\right] ^{2} }. \end{aligned}$$

At the limit $\tau \rightarrow 0$, we have

73 $$\begin{aligned} \lim _{ \tau \rightarrow 0} E_n'= E_n=\frac{\pi ^2\hbar ^2n^2}{2m_0a^2}. \end{aligned}$$

Thus, the energy levels can be rewritten as

74 $$\begin{aligned} E_n=\frac{3}{4} \left[ \frac{\tau L}{\arctan \left( \frac{2\tau L-1}{\sqrt{3}}\right) +\frac{\pi }{6} }\right] ^2 E_n<E_n. \end{aligned}$$

The effects of the parameter $\tau$ in $y_s$ direction induce deformations of quantum levels, which consequently lead to a decrease in the amplitude of the energy levels. The corresponding wave functions to the energies (72) are given by

75 $$\begin{aligned} \psi _n (x) = \frac{B}{\sqrt{1-\tau y_s+\tau ^2 y_s^2}}\sin \left( \frac{ n\pi }{\left[ \arctan \left( \frac{2\tau a-1}{\sqrt{3}}\right) +\frac{\pi }{6}\right] ^{2}}\left[ \arctan \left( \frac{2\tau y_s-1}{\sqrt{3}}\right) +\frac{\pi }{6}\right] \right) . \end{aligned}$$

The total eigenvalues of the system are given by

76 $$\begin{aligned} E_n^t= & {} n^2\frac{\pi ^2\hbar ^2}{2m_0a^2}+ \frac{3\pi ^2 \tau ^2\hbar ^2n^2}{8m_0 \left[ \arctan \left( \frac{2\tau a-1}{\sqrt{3}}\right) +\frac{\pi }{6}\right] ^{2} },\nonumber \\= & {} \left( 1+\frac{3\tau ^2 a}{\left[ \arctan \left( \frac{2\tau a-1}{\sqrt{3}}\right) +\frac{\pi }{6}\right] ^{2}}\right) E_n, \end{aligned}$$

and

77 $$\begin{aligned} \lim _{ \tau \rightarrow 0} E_n^t =2E_n, \end{aligned}$$

The wave function in $x_s,y_s$-directions are given by

78 $$\begin{aligned} \psi (x_s,y_s)= & {} \frac{B\sqrt{\frac{2}{a}}}{\sqrt{1-\tau y_s+\tau ^2 y_s^2}} \sin \left( \frac{n\pi }{a}x_s\right) \nonumber \\&\times \sin \left( \frac{ n\pi }{\left[ \arctan \left( \frac{2\tau a-1}{\sqrt{3}}\right) +\frac{\pi }{6}\right] ^{2}}\left[ \arctan \left( \frac{2\tau y_s-1}{\sqrt{3}}\right) +\frac{\pi }{6}\right] \right) . \end{aligned}$$

At the limit $\tau \rightarrow 0$, we have

79 $$\begin{aligned} \lim _{ \tau \rightarrow 0} \psi (x_s,y_s)= \frac{2}{a} \sin \left( \frac{n\pi }{a}x_s\right) \sin \left( \frac{n\pi }{a}y_s\right) . \end{aligned}$$

The corresponding probability density is given by

80 $$\begin{aligned} \rho (x_s,y_s)= & {} \frac{2B^2}{a(1-\tau y_s+\tau ^2 y_s^2)} \sin ^2\left( \frac{n\pi }{a}x_s\right) \nonumber \\&\times \sin^2 \left( \frac{ n\pi }{\left[ \arctan \left( \frac{2\tau a-1}{\sqrt{3}}\right) +\frac{\pi }{6}\right] ^{2}}\left[ \arctan \left( \frac{2\tau y_s-1}{\sqrt{3}}\right) +\frac{\pi }{6}\right] \right) . \end{aligned}$$

**Figure 8:**
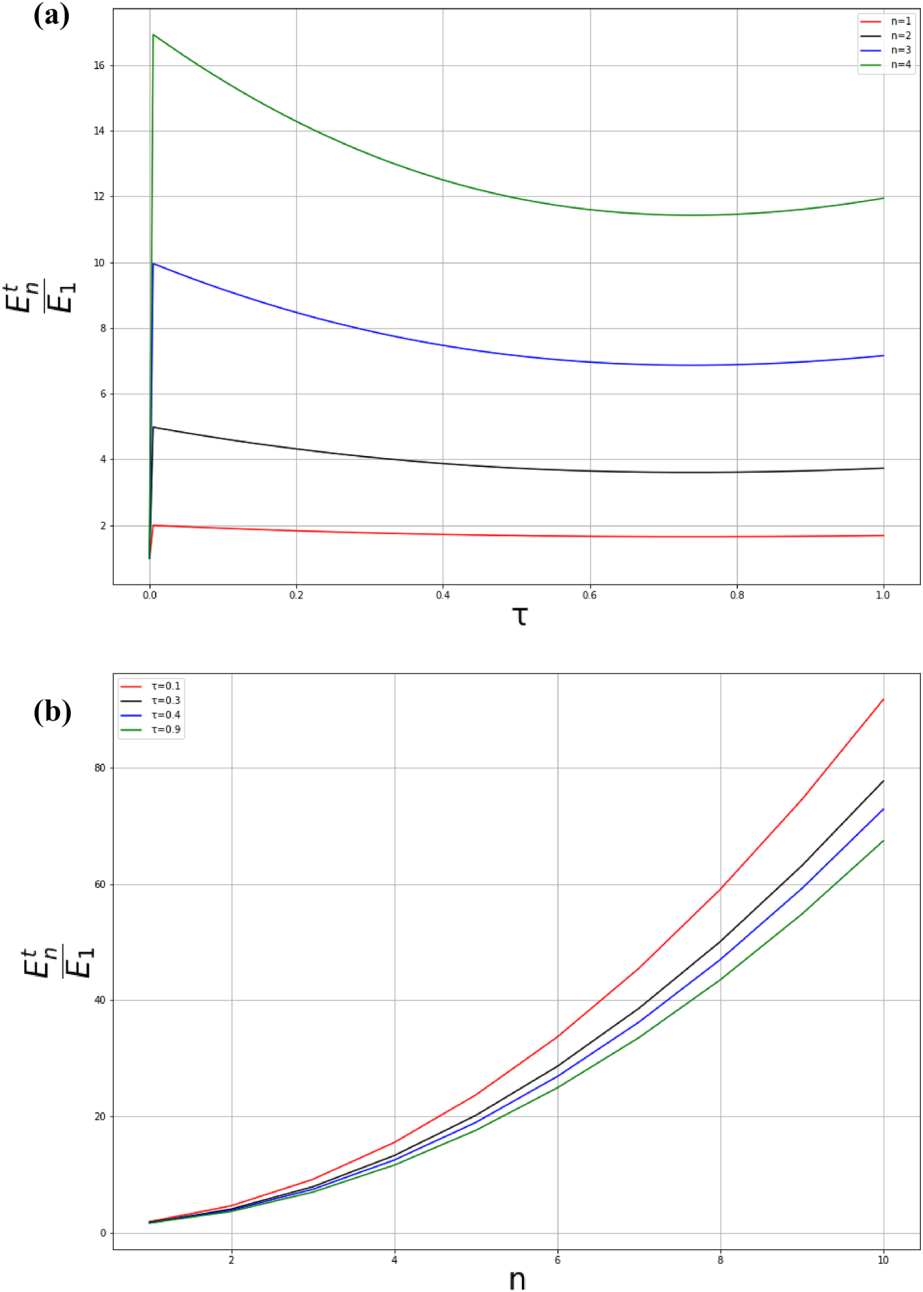
The energy $E_n/E_1$ of the particle in 2D box of length $a = 1$ with mass $m_0 = 1$ and $\hbar = 1$.

Figure 8 illustrates the energy levels of the particle as functions of the quantum number *n* and the quantum gravitational parameter $\tau$. As one increases the quantum number *n* from the fundamental level, the increase in $\tau$ gradually curves the energy levels (Figure (a)). For fixed values of $\tau$, Figure (b) illustrates energy levels versus the quantum number *n*. Conversely to the graph obtained in^68,73–75^, one can see that, when $\tau$ increases, the amplitudes of energy levels $E_n^t/E_1$ decrease. In fact, by increasing the quantum gravitational effects, it leads to the enhancement of binding quantum levels allowing particles to jump from one state to another with low energies^13^.

**Figure 9:**
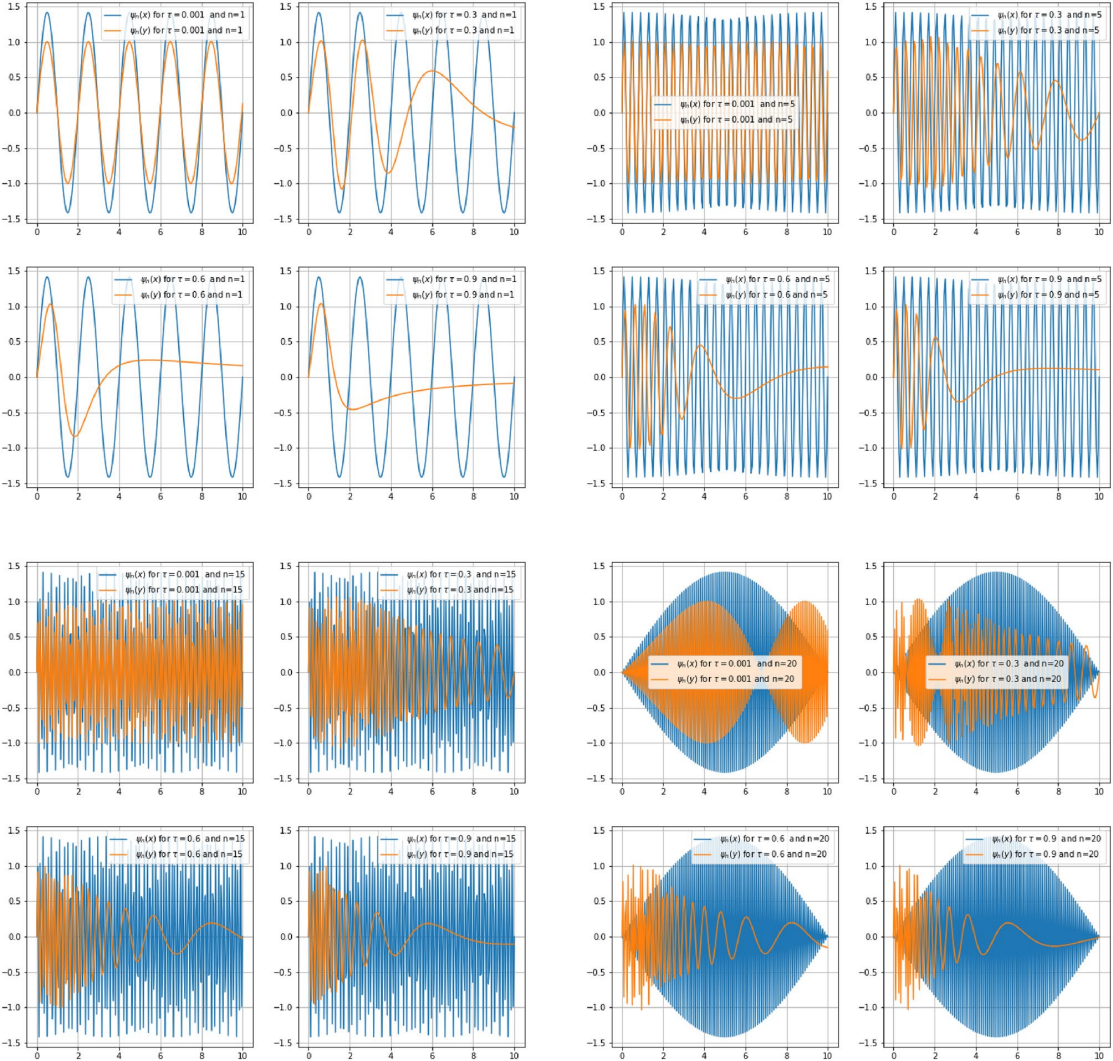
Comparison graph of $\psi _n(x_s)$ and $\psi _n(y_s)$ for a particle confined in an infinite square well of length $a = 1$ deformed by the gravity parameter $\tau$ in ys direction.

Figure 9 illustrates a comparison between eigenfunctions $\psi _n(x_s)$ and $\psi _n(y_s)$ for fixed values of *n* and $\tau$. The wave function in $x_s$-direction is taken as a witness with respect to that of the $y_s$-direction where the effects of quantum gravity are strongly applied. For $n\in \{1;5;15;20\}$, $\psi (y_s)$ compresses and contracts inward as one increases $\tau$. This fact comes to confirm the fundamental property of gravity which is length contraction.

**Figure 10:**
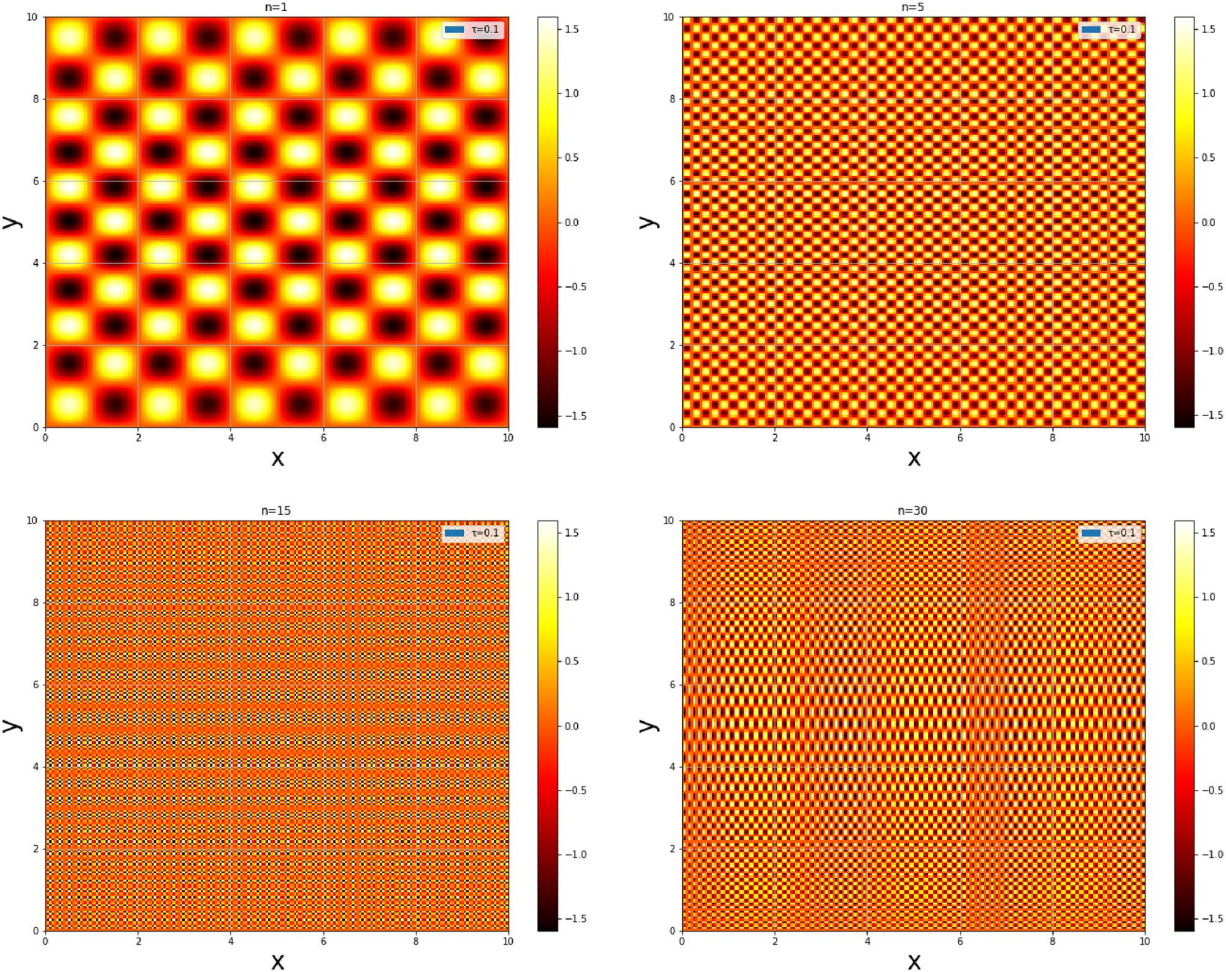
The probability density of a two-dimensional infinite square well for $\tau = 0.1$.

Unlike the figure reported in citations^73,74^, Fig. 10 depicts probability density plots for the three lower states $n = 1; n = 5; n = 15; n = 30$ for a fixed value of $\tau$ ($\tau = 0.1)$, it can be seen that the probability of finding a particle is practically the same everywhere in the square well and this probability strongly increases with the quantum number. This indicates that the deformations allow particles to jump from one state to another with low energies and with high probability densities.

## Concluding remarks

In this paper, we revisited our recent concept of minimal and maximal lengths^12^ (previously predicted by Perivolaropoulos^11^) in the context of non-Hermitian position-dependent noncommutativity. We have shown that the existence of both lengths has interesting and significant properties of quantum gravity at the Planck scale. We showed that measuring both lengths at the same time produces a lattice system with each site represented by a minimal length $l_{min}$ separated by a maximal length $l_{max}$. At each singular point, $l_{min}$ results from the unification of strong magnetic and weak quantum gravitational fields. Furthemore, we have demonstrated that the maximal length of quantum gravity at this scale manifests properties similar to classical gravity and allows probing quantum gravitational effects with low energies. Moreover the existence of minimal uncertainties lead almost unavoidably to non-Hermicity of some operators that generate the noncommutative algebra (1). Consequently, Hamiltonians of systems involving these operators will not be Hermitian and the corresponding Hilbert space structure is modified. In order to map these operators into Hermitian ones, we have introduced an appropriate Dyson map and by means of a similarity transformation, we have generated a hidden Hermitian position-dependent noncommutativity (14). Furthemore, we have demonstrated that the maximal length of quantum gravity at this scale manifests properties similar to classical gravity and allows probing quantum gravitational effects with low energies. Moreover the existence of minimal uncertainties lead almost unavoidably to non-Hermicity of some operators that generate the noncommutative algebra (1). Consequently, Hamiltonians of systems involving these operators will not be Hermitian and the corresponding Hilbert space structure is modified. In order to map these operators into Hermitian ones, we have introduced an appropriate Dyson map and by means of a similarity transformation, we have generated a hidden Hermitian position-dependent noncommutativity (14). This transformation preserves the properties of quantum gravity but removes the fuzziness caused by the minimal uncertainties. Finally, to find the representation of a free particle in this new space, we have solved a non-linear Schrödinger equation. To do so, we have transformed this equation into von Roos equation^67^, then by an appropriate change of variable, we reduced this equation into a simple and solvable non-linear Schrödinger equation. We observed that the increase of quantum gravitational effects $\tau$ in this region curves the quantum energy levels. These curvatures are more pronounced as one increases the quantum levels, allowing the particle to jump from one state to another with low energies and with high probability densities. Furthermore, they contract and compress the wave function in $y_s$-direction.

However, one can wonder about what happens in the case of a harmonic oscillator? In this way, the Hamiltonian of the system is given by

81 $$\begin{aligned} {\hat{H}}_{ho}=\frac{1}{2m_0}\left( {\hat{P}}_x^2+{\hat{P}}_y^2\right) +\frac{1}{2}m_0\omega ^2({\hat{X}}^2+{\hat{Y}}^2). \end{aligned}$$

In terms of the $\theta$-deformed variables, this Hamiltonian can also be re-written as follows

82 $$\begin{aligned} {\hat{H}}_{ho}= & {} \frac{1}{2m_0}\left[ {\hat{p}}_{x_0}^2+\left( 1-\tau {\hat{y}}_0+\tau ^2 {\hat{y}}_0^2\right) ^2 {\hat{p}}_{y_0}^2-i\hbar \tau (1+2\tau {\hat{y}}_0)(1-\tau {\hat{y}}_0 +\tau ^2{\hat{y}}_0^2){\hat{p}}_{y_0} \right] +\nonumber \\&\frac{1}{2}m_0\omega ^2\left[ \left( 1-\tau {\hat{y}}_0+\tau ^2 {\hat{y}}_0^2\right) ^2 {\hat{x}}_0^2-i\theta (1-2\tau y_0)\left( 1-\tau {\hat{y}}_0+\tau ^2 {\hat{y}}_0^2\right) x_0+y_0^2\right] . \end{aligned}$$

Since this Hamiltonian ${\hat{H}}_{ho}$ is evidently non-Hermitian, we have to employ a Dyson map to convert it into Hermitian one as in the previous example

83 $$\begin{aligned} {\hat{h}}_{ho}=\eta {\hat{H}}_{ho}\eta ^{-1}= \frac{1}{2m_0}\left( {\hat{p}}_x^2+{\hat{p}}_y^2\right) +\frac{1}{2}m_0\omega ^2({\hat{x}}^2+{\hat{y}}^2). \end{aligned}$$

This Hamiltonian may be also re-expressed as follows using the representations (10,11,12,13)

84 $$\begin{aligned} {\hat{h}}_{ho}= & {} \frac{1}{2m_0}\left[ {\hat{p}}_{x_0}^2+ (1-\tau {\hat{y}}_0 +\tau ^2 {\hat{y}}_0^2)^{1/2}{\hat{p}}_{y_0} (1-\tau {\hat{y}}_0 +\tau ^2 {\hat{y}}_0^2) {\hat{p}}_{y_0} (1-\tau {\hat{y}}_0 +\tau ^2 {\hat{y}}_0^2)^{1/2}\right] +\nonumber \\&\frac{1}{2}m_0\omega ^2 \left[ {\hat{y}}_0^2+ (1-\tau {\hat{y}}_0 +\tau ^2 {\hat{y}}_0^2)^{1/2}{\hat{x}}_0 (1-\tau {\hat{y}}_0 +\tau ^2 {\hat{y}}_0^2) {\hat{x}}_0 (1-\tau {\hat{y}}_0 +\tau ^2 {\hat{y}}_0^2)^{1/2}\right] . \end{aligned}$$

The eigensystems of the Eq. (84) are far more complicated to obtain with the same method as in the previous models, as the system viewed as a differential equation no longer decouples in $x_0$ and $y_0$. We leave the construction of solutions for this model by alternative means to future work.

## References

[1] Seiberg N, Witten E (1999). String theory and noncommutative geometry. JHEP.

[2] Amati D., Ciafaloni M., Veneziano G. (1989). Can Space-Time Be Probed Below the String Size?. Phys. Lett B.

[3] Scardigli F (1999). &nbsp;Generalized uncertainty principle in quantum gravity from microblack hole gedanken experiment,. Phys.Lett. B.

[4] Rovelli C, Smolin L (1995). Discreteness of area and volume in quantum gravity. Nucl. Phys. B..

[5] Kempf A, Mangano G, Mann R. (1995). Hilbert space representation of the minimal length uncertainty relation. Phys. Rev D.

[6] Kempf A, Mangano G (1997). Minimal length uncertainty relation and ultraviolet regularization. Phys. Rev. D.

[7] Kempf A (1994). Uncertainty relation in quantum mechanics with quantum group symmetry. J. Math Phys.

[8] Nozari K., Etemadi A. (2012). Minimal length, maximal momentum and Hilbert space representation of quantum mechanics. Phys. Rev D.

[9] Pedram P (2012). A higher order GUP with minimal length uncertainty and maximal momentum,. Phys. Lett B.

[10] Pedram P (2012). A higher order GUP with minimal length uncertainty and maximal momentum II. J. Phys. Lett B.

[11] Perivolaropoulos L (2017). Cosmological horizons, uncertainty principle, and maximum length quantum mechanics. Phys. Rev.

[12] Lawson L. (2020). Minimal and maximal lengths from position-dependentnoncommutativity. J. Phys. A: Math. Theor.

[13] Lawson L (2022). Position-dependent mass in strong quantum gravitational background fields. J. Phys. A Math. Theor..

[14] Tawfik A, Diab A (2015). A review of the generalized uncertainty principle. Rep. Prog. Phys..

[15] Lambiase G, Scardigli F (2018). Lorentz violation and generalized uncertainty principle. Phys. Rev. D.

[16] Kempf A (1994). Quantum field theory with nonzero minimal uncertainties in positions and momenta. Czech. J. Phys..

[17] Kanazawa T, Lambiase G, Vilasi G, Yoshioka A (2019). Noncommutative Schwarzschild geometry and generalized uncertainty principle. Eur. Phys. J. C.

[18] Ong Y (2018). Generalized uncertainty principle, black holes, and white dwarfs: A tale of two infinities. J. Cosmol. Astropart. Phys. JCAP.

[19] Jana T, Roy P (2009). Non-Hermitian quantum mechanics with minimal length uncertainty. SIGMA.

[20] Bagchi B, Fring A (2009). Minimal length in Quantum mechanics and nonHermitian Hamiltonian systems. Phys. Lett. A.

[21] Dey S, Fring A, Khantoul B (2013). Hermitian versus non-Hermitian representations for minimal length uncertainty relations. J. Phys. A Math. Theor..

[22] Dyson F (1956). Thermodynamic behavior of an ideal ferromagnet. Phys. Rev..

[23] Dey S, Fring A, Gouba L (2014). PT-symmetric noncommutative spaces with minimal volume uncertainty relations. J. Phys. A Math. Theor..

[24] Fring A, Gouba L, Bagchi B (2010). Minimal areas from q-deformed oscillator algebras. J. Phys. A Math. Theor..

[25] Fring A, Gouba L, Scholtz F (2010). Strings from position-dependent noncommutativity. J. Phys. A Math. Theor..

[26] Lawson L, Gouba L, Avossevou G (2017). Two-dimensional noncommutative gravitational quantum well. J. Phys A Math. Theor.

[27] Alavi S, Abbaspour S (2014). Dynamical noncommutative quantum mechanics. J. Phys. A Math. Theor..

[28] Dey S, Fring A (2013). The two dimensional harmonic oscillator on a noncommutative space with minimal uncertainties. Acta Polytech..

[29] Lawson L, Nonkané I, Sodoga K (2021). The damped harmonic oscillator at the classical limit of the Snyder-de Sitter space. J. Math. Res..

[30] Gomes M, Kupriyanov V (2009). Position-dependent noncommutativity in quantum mechanics. Phys. Rev. D.

[31] Kupriyanov V (2013). Quantum mechanics with coordinate dependent noncommutativity. J. Math. Phys..

[32] Kupriyanov V (2013). A hydrogen atom on curved noncommutative space. J. Phys. A.

[33] Bender C (2007). Making sense of non-Hermitian Hamiltonians. Rep. Prog. Phys..

[34] Mostafazadeh A (2002). Pseudo-Hermiticity versus PT symmetry. The necessary condition for the reality of the spectrum. J. Math. Phys..

[35] Mostafazadeh A (2002). Pseudo-Hermiticity versus PT-symmetry. II. A complete characterization of non-Hermitian Hamiltonians with a real spectrum. J. Math. Phys..

[36] Mostafazadeh A (2002). Pseudo-Hermiticity versus PT-symmetry III. Equivalence of pseudo-Hermiticity and the presence of antilinear symmetries. J. Math. Phys..

[37] Scholz F, Geyer H, Hahne F (1992). Quasi-Hermitian operators in quantum mechanics and variational principle. Ann. Phys..

[38] Dieudonné, J. Quasi-Hermitian operators. In *Proceedings of the International Symposium on Linear Spaces*, Jerusalem 1960, 115-122 (Pergamon, Oxford, 1961)

[39] Froissart M (1959). Covariant formalism of a field with indefinite metric. II Nuovo Cimento.

[40] Sudarshan E (1961). Quantum mechanical systems with indefinite metric. I. Phys. Rev..

[41] Mostafazadeh A (2010). Pseudo-Hermitian Representation of Quantum Mechanics. Int. J. Geom. Meth. Mod. Phys..

[42] Znojil M (2008). Time-dependent version of cryptohermitian quantum theory. Phys. Rev. D.

[43] Znojil M (2009). Three-Hilbert-space formulation of quantum mechanics. SIGMA.

[44] Smilga A (2008). Cryptogauge symmetry and cryptoghosts for crypto-Hermitian Hamiltonians. J.Phys. A.

[45] Santos J, Luiz F, Duarte O, Moussa M (2019). Non-Hermitian noncommutative quantum mechanics. Eur. Phys. J. Plus.

[46] Swanson M (2004). Transition elements for a non-Hermitian quadratic Hamiltonian. J. Math. Phys..

[47] Kempf A. (1997). Noncommutative geometric regularization. Phys. Rev D.

[48] Kempf A (1997). Maximal localization in the presence of minimal uncertainties in positions and in momenta. Phys. Rev. D..

[49] Kempf, A., & Mangano, G., Minimal length uncertainty relation and ultraviolet regularization. *Phys. Rev. D.* **55**, 7909–7920 (1997)

[50] Szabo R (2003). Quantum field theory on noncommutative spaces. Phys. Rept.

[51] Muller-Hoissen, F., Noncommutative geometries and gravity, in Recent Developments in Gravitation and Cosmology.* AIP Conf. Proc., * 977, Amer. Inst. Phys., Melville, NY, 2008, 12–29, arXiv:0710.4418

[52] Delduc F, Duret Q, Gieres F (2008). Magnetic fields in noncommutative quantum mechanics. J. Phys: Conf. Ser.

[53] Szabo R (2003). Quantum field theory on noncommutative spaces. Phys. Reports.

[54] Bigatti D, Susskind S (2000). Magnetic fields, branes and noncommutative geometry. Phys. Rev D.

[55] Mtchedlidze, S. *et al.* Evolution of primordial magnetic fields during large-scale structure formation, arXiv:2109.13520 [astro-ph.CO] (2021)

[56] kempf A (2000). Unsharp degrees of freedom and the generating of symmetries. Phys. Rev. D.

[57] Scardiglia F, Casadio R (2015). Gravitational tests of the Generalized Uncertainty Principle. Eur. Phys. J. C.

[58] Lambiase G, Scardigli F (2018). Lorentz violation and generalized uncertainty principle. Phys. Rev D.

[59] Pedram P (2012). A higher order GUP with minimal length uncertainty and maximal momentum. Phys. Lett. B.

[60] Pedram P. (2012). A higher order GUP with minimal length uncertainty and maximal momentum II. Phys. Lett B.

[61] Tawfik A (2015). A review of the generalized uncertainty principle. Rep. Prog. Phys.

[62] Sabri Y., Nouicier K (2012). Phase transitions of a GUP-corrected Schwarzschild black hole within isothermal cavities. Class. Quant Grav.

[63] Ali, A., Das, S., & Vagenas, E., Discreteness of space from the generalized uncertainty principle.* Phys. Lett.B* **678**, 497 (2009)

[64] Das, S., Vagenas, E. & Ali, A., Discreteness of space from GUP II: Relativistic wave equations. *Phys. Lett. B*, **690,** 407 (2010)

[65] Pedram, P., A higher order GUP with minimal length uncertainty and maximal momentum. *Phys. Lett. B* **714,** 317–323 (2012)

[66] Landau L (1927). Diamagnetismus der Metalle. Phys. Rev. A.

[67] von Roos O (1983). Position-dependent effective masses in semiconductor theory. Phys. Rev. B.

[68] da Costa GB, Gomez I, Portesi M. (2020). κ-Deformed quantum and classical mechanics for a system with position-dependent effective mass. J. Math. Phys..

[69] Gazeau, J., Coherent States in Quantum Physics, (Wiley-Vch Verlag Gmbh Co.KgaA, 2009)

[70] Perelomov, A. Generalized Coherent States and Their Applications (Springer-Verlag, Heidelberg) 1986

[71] Klauder, J. and Skagerstam, B., Coherent States: Applications in Physics and Mathematical Physics (World Scientific) 1985;

[72] Ali ST, Antoine JP, Gazeau JP (2000). Coherent States, Wavelets and Their Generalizations.

[73] Costa Filho, R.., Almeida, M., Farias, G., and Andrade Jr., J., Displacement operator for quantum systems with position-dependent mass. *Phys Rev A,* **84**, 050102 (2011).

[74] Habib Mazharimousavi S. (2012). Revisiting the displacement operator for quantumsystems with position-dependent mass. Phys. Rev. A.

[75] Bruno da Costa G.B., & Borges, E.P. A position-dependent mass harmonic oscillator and deformed space. *J. Math. Phys.*** 59,** 042101 (2018)

